# Generation of Aggregates of Mouse Embryonic Stem Cells that Show Symmetry Breaking, Polarization and Emergent Collective Behaviour *In Vitro*

**DOI:** 10.3791/53252

**Published:** 2015-11-24

**Authors:** Peter Baillie-Johnson, Susanne Carina van den Brink, Tina Balayo, David Andrew Turner, Alfonso Martinez Arias

**Affiliations:** ^1^Department of Genetics, University of Cambridge; ^2^Hubrecht Institute, Royal Netherlands Academy of Arts and Sciences

**Keywords:** Developmental Biology, Issue 105, Mouse, gastrulation, self-organization, symmetry breaking, polarization, axial elongation, live-cell imaging, gastruloids

## Abstract

We have developed a protocol improving current Embryoid Body (EB) culture which allows the study of self-organization, symmetry breaking, axial elongation and cell fate specification using aggregates of mouse embryonic stem cells (mESCs) in suspension culture. Small numbers of mESCs are aggregated in basal medium for 48 hr in non-tissue-culture-treated, U-bottomed 96-well plates, after which they are competent to respond to experimental signals. Following treatment, these aggregates begin to show signs of polarized gene expression and gradually alter their morphology from a spherical mass of cells to an elongated, well organized structure in the absence of external asymmetry cues. These structures are not only able to display markers of the three germ layers, but actively display gastrulation-like movements, evidenced by a directional dislodgement of individual cells from the aggregate, which crucially occurs at one region of the elongated structure. This protocol provides a detailed method for the reproducible formation of these aggregates, their stimulation with signals such as Wnt/β-Catenin activation and BMP inhibition and their analysis by single time-point or time-lapse fluorescent microscopy. In addition, we describe modifications to current whole-mount mouse embryo staining procedures for immunocytochemical analysis of specific markers within fixed aggregates. The changes in morphology, gene expression and length of the aggregates can be quantitatively measured, providing information on how signals can alter axial fates. It is envisaged that this system can be applied both to the study of early developmental events such as axial development and organization, and more broadly, the processes of self-organization and cellular decision-making. It may also provide a suitable niche for the generation of cell types present in the embryo that are unobtainable from conventional adherent culture such as spinal cord and motor neurones.

**Figure:**
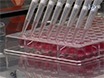


## Introduction

The study and understanding of cell-fate decisions in early mammalian development can make use of cultures of Embryonic Stem Cells (ESCs), clonal populations derived from blastocysts which have the ability to self renew and differentiate into all cell types of an organism *i.e*., they are pluripotent^1,2^. While these cultures have been and are continuing to be useful for the understanding of the molecular basis of cell-fate decisions, they are unable to reproduce some of the spatial arrangements and global behaviours that are generated in embryos during gastrulation. In the embryo, the process of gastrulation transforms a single epithelial layer into the three distinct germ layers and endows the embryo with an overt anteroposterior organization^3-5^. Attempts to recapitulate these events *ex vivo* have been based on the generation of three-dimensional aggregates of ESCs, referred to as embryoid bodies (EBs), and subjecting them to differentiation conditions^6,7^. These aggregates can be coaxed to differentiate into many different cell types, some of which are either unable to be obtained or induced with low efficiency in adherent culture or cannot be produced at all; *e.g*., blood^8^ and primordial germ cells^9^. A limitation in the use of EBs however, is that they are unable to display the morphogenetic behaviour, germ layer distribution or axial organization that are major characteristics of the developing embryo, resulting in spatial disorganization^6,10^. In one report, treatment of EBs with Wnt leads to a weak polarization in gene expression in some aggregates but no clear morphogenesis is observed^7^. In more recent reports, EBs that have been cultured for extended periods of time develop anterior structures such as retinas, cortex and inner ear sensory cells, which mimic their embryonic counterparts but develop without the context of an axial organization^11-13^.

A report by Marikawa *et al.*^14^, whilst working with aggregates of mouse P19 Embryo Carcinoma (EC) cells formed by the hanging-drop method, reported the emergence of elongated structures of mesodermal origin reminiscent of the elongations that are observed with exogastrulae in amphibian and sea urchin embryos and Keller explants^15-18^. As this had not been observed with mouse embryonic stem cells (mESCs), we attempted to reproduce the behaviour observed with P19 EC cells using aggregates of mESCs^19^ and we report culture conditions that lead to their symmetry breaking and axial elongation. An important difference from the work with P19 cells is that the aggregation protocol described here is performed in a 96-well plate, similar to that described by Eiraku *et al*.^20^, instead of hanging drops. This change resulted in an increased efficiency in terms of aggregate recovery and in both intra- and inter-experimental reproducibility. Importantly, maintaining aggregates in individual wells ensures that fusion between aggregates (common when pooling aggregates from hanging drops) does not occur. In addition, a key feature of the protocol is 24 hr exposure to the GSK3 inhibitor CHI99021 (Chi), a potent activator of Wnt/β-Catenin signalling, following aggregation.

The method described here provides a basis for understanding the processes of self-organization, axial organization and germ layer specification in culture, allowing inferences to be made concerning axial development *in vivo*^19,21^. For these reasons the method has the potential to allow detailed mechanistic analysis of processes that may be difficult to study in the embryo. Furthermore, a potential application is in the generation of tissues and organs that are not easily obtainable in adherent culture due to the lack of a structured cellular niche such as spinal cord precursors^21^ and motor neurones. Three-dimensional aggregate culture offers a physical structure and signalling environment that may not be achievable by conventional means, leading to a new approach for the derivation of embryonic lineages in a spatially organized manner.

## Protocol

### 1. Culture Conditions Prior to Aggregation

1. Maintain mESCs in ESLIF medium (see Table of Specific Materials & Equipment for formulation) on gelatin-coated 25 cm^2^ tissue-culture-treated flasks in a humidified incubator at 37 °C and 5% CO_2_^21-25^.
2. Grow cells for at least two passages post thawing before use in this protocol; cells at lower passages are generally more successful at generating reproducible characteristics throughout experimental replicates (*i.e*., no more than 15 passages in culture), however slight variation between different ES cell-lines is to be expected (see **Table 2** for the cell lines tested).
3. Grow cells to 40-60% confluency. Do not use over-confluent cells for aggregation.

### 2. Generation of Aggregates

1.  Pre-warm PBS (+Ca^2+^, +Mg^2+^), ESLIF, N2B27^26,27^ and Trypsin-EDTA in a 37 °C water bath.
2.  Aspirate culture medium from the tissue culture flask (step 1.3). Rinse the flask gently with 5 ml PBS, twice. Aspirate the PBS and add 1 to 2 ml of pre-warmed Trypsin-EDTA (0.25%) to dissociate the cells.
  1.  Place the flask in the incubator for < 5 min or until cells have fully detached from the surface of the flask. Pipette up and down with a 1 ml pipette to generate single-cell suspension for accurate counting and uniform aggregate size formation.
3.  Neutralize Trypsin with 5-10 ml ESLIF; wash down the growth surface to maximize cell recovery and transfer to a 50 ml centrifuge tube. Remove a 1 ml aliquot from the centrifuge tube and count the cells with a haemocytometer.
4.  Determine the volume of suspension required to give 10 cells/µl (a whole 96-well plate requires 5x10^4^ cells in a 5 ml suspension). Count cells accurately, as large deviations from the stated cell number can adversely affect the response of the aggregates to stimuli.
5.  Add 5x10^4^ cells to 5 ml pre-warmed PBS in a fresh 50 ml centrifuge tube and spin at ~170 x g for 5 min.
6.  Carefully aspirate the PBS and add 5 ml pre-warmed PBS gently; do not disturb the pellet at the bottom of the tube. Centrifuge at ~170 x g for 5 min.
7.  Carefully aspirate PBS for a second time. Remove as much PBS as possible without disturbing the pellet as PBS carryover can adversely affect aggregation. Resuspend the pellet firstly in 1 ml warm N2B27 with a P1000 pipette to generate a homogenous cell suspension, followed by further addition of N2B27 to the required volume (*e.g.,* add 4 ml for a 5x10^4^ cells/5 ml suspension).
8.  Transfer the cell suspension to a sterile reservoir and pipette a 40 µl droplet into the bottom of each well of a non-tissue-culture treated, ‘U’-bottomed 96-well plate using a multichannel pipette. Cover the 96-well plate with its corresponding lid and confirm the presence of cells with an inverted bench-top microscope (**Figure 1B**). Note: It is essential that these plates are used to limit the possibility of cells adhering. Do not coat the bottom of the 96-well plate with Gelatin, Fibronectin or any other coating that promotes cell adhesion.
9.  Incubate the cells for 48 hr in a humidified incubator at 37 °C and 5% CO_2_ for aggregation.

### 3. Applying Stimuli and Changing Medium

1.  Following the 48 hr incubation period, observe the appearance of the mESCs within each well of the 96-well plate to confirm successful aggregation (**Figure 1E**). Note: Aggregates will appear spherical in nature and approximately 150-200 µm in diameter. Refer to the troubleshooting section if problems arise (**Table 1**).
2.  Add 150 µl fresh secondary medium (3 µM Chi99021 (Chi) in N2B27^19^; stock prepared at 10 mM in DMSO) to each well using a multichannel pipette. Pipette with enough force to dislodge any aggregates that may have started to adhere to the bottom of the wells. Incubate aggregates in secondary medium for 24 hr in a humidified incubator at 37 °C and 5% CO_2_ (**Figure 1A**). Note: Reproducible elongated and polarized aggregates are generated using this secondary medium. Other secondary medium compositions may also be used depending on the experimental conditions required and examples are shown in **Figure 3**.
3.  For subsequent medium changes, use a multichannel pipette held at an angle (approximately 30°) to gently remove 150 µl of the secondary medium from the side of each well. Then, pipette 150 µl fresh N2B27 into each well with enough force to dislodge any aggregates that may have started to adhere to the bottom of the wells.
4.  Repeat point 3. 3 every 24 hr until the time-course is complete (the typical length of an aggregate culture experiment is 120 hr). Note: Ensure aggregates are freely moving following medium changes to ensure reproducibility and consistency within and between each 96-well plate. Medium must be changed daily following the aggregation period.

### 4. Preparation of Aggregates for Immunostaining and Analysis by Confocal Microscopy

1. Fixation and Primary Antibody Incubation Note: The protocol described by A.K. Hadjantonakis^28^ has been modified to suit immunostaining of mESC aggregates. For the typical antibodies used in our studies and their dilutions, refer to previously published work^19,21,24,25,29^ and **Table 3**.
  1. Use a multichannel pipette held at an angle (approximately 30°) to gently remove 150 µl medium from the side of each well of the 96-well plate. Wash twice by pipetting 150 µl PBS into each well. Leave a couple of minutes between each wash to allow the aggregates to settle.
  2. Set a P1000 pipette to dispense 200 µl, attach the corresponding pipette tip and cut off approximately 3 mm from the end of the tip with a pair of sterile scissors. Draw a portion of the PBS within each well of the 96-well plate a short way into the tip and expel it to agitate the aggregate.
  3. Use the same tip to draw up the whole volume within the well including the aggregate and pipette into a small (30 mm diameter) glass *Drosophila* dissection well. Transfer all the aggregates from the 96-well plate that will undergo identical immunostaining regimes into the same glass dissection well. Note: Use a new cut pipette tip when transferring aggregates from different experimental conditions to prevent carryover of aggregates.
  4. Place the glass well that contains the aggregates onto a dissection microscope. Swirl the glass well to force the aggregates to the centre and aspirate PBS from one side of the well with a glass Pasteur pipette. Use the microscope to ensure the aggregates are not aspirated. Leave a small volume of PBS within the glass well to cover the aggregates to prevent them from drying out.
  5. Add 1 ml freshly prepared formaldehyde (4%) dissolved in PBS and incubate for 2 hr at 4 °C on an orbital shaker set to a low speed. Caution: Paraformaldehyde is known to be allergenic, carcinogenic and toxic. Wear appropriate protection whilst handling.
  6. Following fixation, aspirate the formaldehyde solution in the same manner as described in section 4. 1. 4 and wash the aggregates with 1 ml PBS three times, 10 min for with each wash, on an orbital shaker set to a low speed.
  7. Aspirate the PBS as described in section 4. 1. 4 and perform a further three, 10 min washes with PBS containing 10% Foetal Bovine Serum and 0.2% Triton X-100 (PBSFT). Perform the 10 min washes on an orbital shaker set to a low speed. Note: Using Foetal Bovine Serum (FBS) results in a clear wash buffer, reducing the number of aggregates that would otherwise be lost during the protocol if milk was used. It is important to note that following fixation, immunohistochemical procedures can be performed in various ways other than those detailed below and should be determined and optimized by the investigator.
  8. Block the aggregates for 1 hr at 4 °C in PBSFT on an orbital rocker set to a low speed. The protocol may be paused at this point, and aggregates blocked O/N, with constant agitation on a horizontal gyratory rocker at 4°C.
  9. Aspirate the PBSFT as previously described (see section 4. 1. 4) and incubate the aggregates with 500 µl of the required primary antibody diluted in PBSFT O/N at 4 °C on an orbital shaker set to low speed. Cover with paraffin film to prevent evaporation.
2. Secondary Antibody Incubation
  1. Aspirate the primary antibody solution and wash with 1 ml PBSFT (at 4 °C) in the following manner: twice for 5 min; thrice for 15 min; and four to seven times for 1 hr. Perform each wash step at 4 °C and on an orbital rocker set to low speed. Note: Chill wash medium prior to use. Do not perform the washes on ice as this can cause the aggregates to fragment.
  2. Aspirate the final wash medium and incubate the aggregates with the required secondary antibody in 500 µl PBSFT O/N at 4 °C in the dark on a horizontal gyratory rocker. Add nuclear stain such as Hoechst if required.
3. Mounting and Imaging by Confocal Microcopy
  1. Wash the aggregates as described in Section 4. 1. 4 with 4 °C PBSFT. After the final wash, rinse the aggregates with 1 ml PBS containing 0.2% FBS and Triton X-100 (PBT) in the following manner: twice for 5 min; thrice for 15 min. Perform washes at RT on an orbital rocker protected from light.
  2. Aspirate the medium as previously described (section 4. 1. 4) and incubate for 30 min in the dark with a 1:1 solution of glycerol:PBT (1 ml) followed by a second 30 min incubation with a 7:3 solution of glycerol:PBT (1 ml). Note: Mix the glycerol and PBT solutions at 4 °C using a rotator.
  3. Aspirate the final glycerol:PBT solution and replace with 1 ml mounting medium^24^. The protocol may be paused at this point prior to mounting (if paused, seal the wells with paraffin film and store the staining wells at 4 °C).
  4. Mount the aggregates on microscope slides by pipetting them in 17 µl droplets with a cut P20 tip (**Figure 2**). Fold double-sided tape back on itself four times to generate spacers and attach to each end of the slide. Place the top coverslip (22 mm x 60 mm) upon these spacers (**Figure 2**). Note: it is essential that a cut tip is used at this stage so as not to damage the samples. The spacers prevent the coverslip crushing the aggregates.
  5. Once the aggregates are mounted, image the samples using confocal microscopy using protocols previously described^19,21,24,25^. Once placed on the microscope, leave the slide undisturbed on the stage for a few minutes to allow the aggregates to settle within the mounting medium.

## Representative Results

Appearance of Cells Immediately After Plating and After Aggregation

Immediately after plating, one can observe the presence of single cells within each well of the 96-well plate with a standard inverted tissue-culture microscope (**Figure 1B**). Within 8 hr of plating, these cells will have begun to descend to the bottom of the well due to gravity and will have started to coalesce into discrete clusters (**Figure 1C**). After 24 hr, the coalescing cells will have completed the primary aggregation, and will have compacted into well defined, yet ragged aggregates where individual cells are indistinct from one another (**Figure 1D**). By the end of the second day (48 hr), the aggregates should look ‘clean’, having taken up all the cells within the well (**Figure 1E**); the bottom of the well may have some cells that have been shed from the aggregate at this stage. Providing that the aggregates have been aggregated in N2B27, at this time-point, they should be spherical, approximately 150-200 µm in diameter and freely moving within the well (*i.e*., not adhered to the bottom of the wells). Aggregation in other media (*i.e*., ESLIF or N2B27 with specific factors) has the potential to alter both the initial aggregate morphology and the response to subsequent stimuli (Turner *et al.*, in preparation).

Representative Morphological Changes

Addition of a 24 hr pulse of secondary medium containing Chi between 48 and 72 hr (**Figure 1A, 5A**) generates well defined elongated aggregates that show polarized expression in specific markers of the germ layers^19,21^. Immediately after the Chi pulse (72 hr), the aggregates will begin to shed cells and start to show their response to these signals towards the end of this day (**Figure 3A**). These responses are manifested by changes in gene expression and morphology, both of which are dependent on the treatment that the aggregates have received following the initial 48 hr aggregation period in N2B27 (**Figure 3B**). If only an endpoint analysis is required, the optimum time to image the aggregates is at approximately 96-120 hr. At this point the aggregates will have developed clear morphologies and gene expression patterns (**Figure 3A**), and their size and mass are still low enough so that ejection of medium from P200 pipette is sufficient to dislodge any that may have attached. Failure to prevent attachment of the aggregate to the well will adversely affect aggregate formation (**Figure 5Biv**). The morphologies and gene expression pattern of the aggregates can be altered depending on the treatment. Representative results for sustained or pulsed regimes with either single treatment or combinations of factors are shown for BMP4, Dorsomorphin H1 (BMP receptor inhibitor), ActivinA, SB43 (Activin/Nodal inhibitor), bFGF or PD03 (MEK inhibitor) (**Figure 3B)**.

Aggregate Imaging and Quantitative Image Analysis

Aggregates are amenable to imaging by either widefield microscopy (principally for time-lapse imaging^19^), or fixed and immunostained for confocal imaging^19,21^ (**Figure 2**). The current protocol and imaging description above allows quantitative information to be gained from these aggregates. Following confocal or wide-field image capture, the length and the corresponding gene expression along the diameter or spine of the aggregate (spherical or elongated respectively; **Figure 4A, B**) can be measured. These analyses are also directly applicable to time-lapse images, where one can obtain information of the rate of growth, size and elongation of the aggregates under different conditions (**Figure 4**).


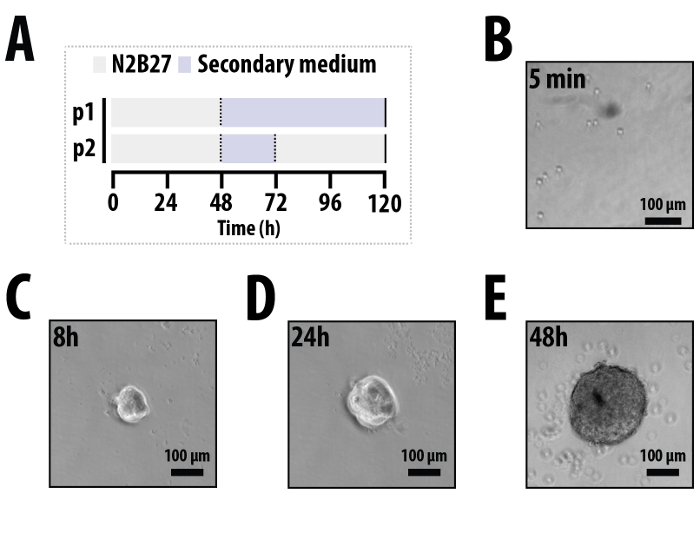
**Figure 1. Typical time-course and early morphologies.(A)** The typical time-course for aggregation experiments. Cells are aggregated for 48 hr in N2B27 containing a suspension of mouse ES cells (10 cells/µl) in a 96-well plate (p1, p2).** (B)** Immediately after plating (t = ~5min), individual cells can be seen in the suspension and have started to form clumps by 8h **(C)**. **(D) **After 24 hr the cells have formed a single aggregate in each well which is approximately 100 µm in diameter. **(E)** After 48 hr aggregation, the aggregate diameter ranges from 150-200 µm. At this point, the aggregation medium (N2B27) is removed and a secondary medium is added either for the rest of the experiment (p1 in part A) or for 24 hr before being changed back to N2B27 (p2 in part A). Scale bar represents 100 µm. Please click here to view a larger version of this figure.


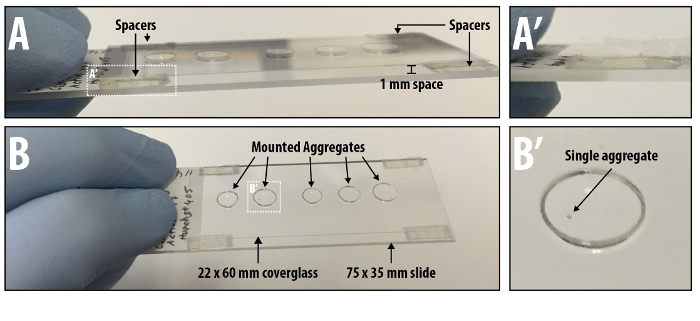
**Figure 2. Mounting aggregates onto microscope slides.** Once the aggregates have been fixed, immunostained and placed in mounting medium, each aggregate is pipetted onto a microscope slide as a 17 µl droplet (A,B,B’). Enough space is left between the aggregate droplets to prevent their merging. Spacers are made with double sided tape and placed at each corner of the microscope slide (A, A’, B). It is upon these that a glass coverslip is placed. Please click here to view a larger version of this figure.


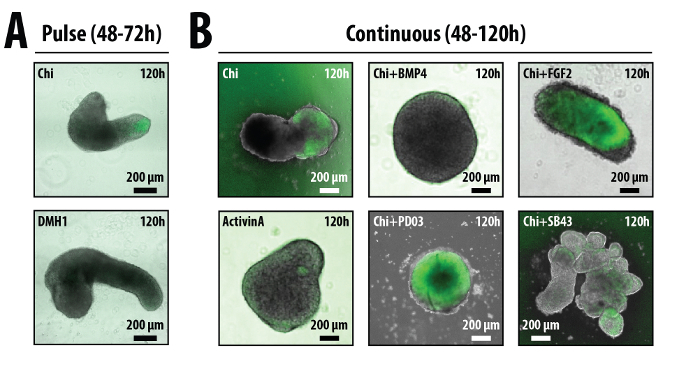
**Figure 3. The effect of different treatments on the morphology of aggregated ES cells.** Following 2 days in N2B27, ES cells have formed aggregates. Addition of specific factors either as a 1 day pulse **(A)** or continuously **(B)** can alter the phenotype of the aggregates with respect to the polarity of gene expression, elongation potential, or their overall shape. The examples in this figure are aggregates formed from either Bra::GFP^30^** (A)** or Sox1::GFP^27^ **(B)** mouse ES cells imaged after 120 hr and treated as indicated. Chi: CHIR 99021^31^; SB43: SB 431542^32^;DM: DorsomorphinH1^33,34^; PD03: PD0325901. Please click here to view a larger version of this figure.


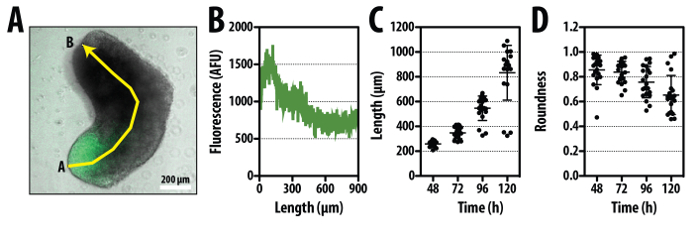
**Figure 4. Quantitative analysis of Aggregates.** A typical aggregate formed from Bra::GFP mESCs^25,30^ following a 24 hr pulse of Chi and imaged at 120 hr. Merged image of the bright-field and GFP channel is shown with segmented line marking the ‘spine’ of the aggregate from point A to B **(A)**, along which the fluorescence and length of the aggregate can be measured **(B)**. The length **(C)** and ‘roundness’ **(D)** from 24 aggregates within the same experiment are shown. Shape descriptors such as the roundness **(D)**, circularity, perimeter and area can be measured using the *Image Analysis Cookbook* plugin from the image analysis software FIJI^35^. Mean indicated by horizontal line at each time-point; error bars indicate standard deviation; scale bar in **(A)** indicates 200 µm. As there are subtle differences between reporter cell lines, it is expected that after a pulse of Chi, the average maximum length and average minimum roundness of the aggregates will vary. Between different cell lines, we find that the average maximum length can be within the range of ~400-800 µm and the average minimum roundness between 0.4 and 0.6. Please click here to view a larger version of this figure.


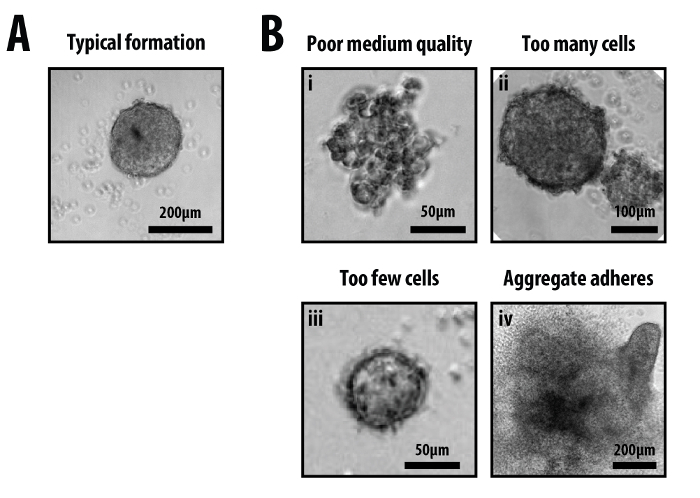
**Figure 5. Examples of failures in aggregate formation.** The ability to generate reproducible aggregates (A) depends on critical factors such as (Bi) fresh and well mixed secondary medium, (Bii, Biii) the accuracy in counting the initial number of cells and (Biv) ensuring the aggregates do not form adherent colonies *i.e*., ‘crash’ into the surface of the well. Typical examples of the errors in aggregate formation for each of the mentioned conditions (B) are shown. Scale-bar as indicated; see trouble-shooting table for details (**Table 1**). Please click here to view a larger version of this figure.


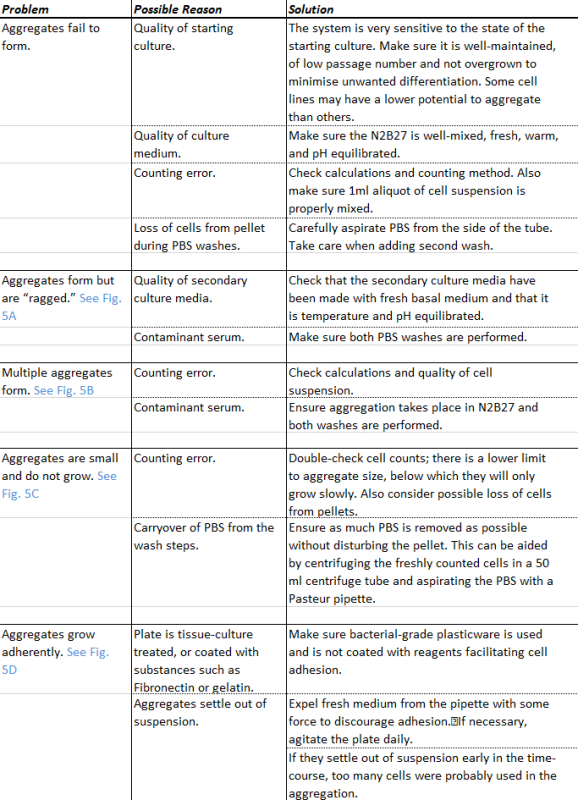
**Table 1. Guide to troubleshooting. **Typical errors associated with the aggregation protocol are given with suggestions as to their resolution.


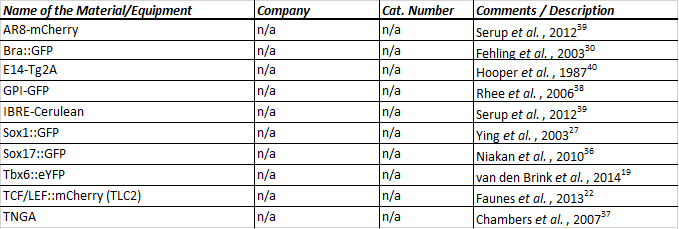
**Table 2. Table of cell lines tested for the formation of aggregates.** A number of cell lines^19,22,27,30,36-40^ have been characterized for the formation of aggregates and, although subtle differences between cell lines are expected and have been observed, all the above lines show similar dynamics in terms of elongation and morphology. In terms of gene expression, the expression pattern is specific to the gene expressed, but within each cell line, the expression pattern is generally consistent over a number of passages. For consistency, we generate aggregates from cells that have not exceeded 15 passages in culture.


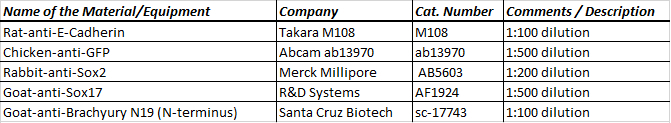
**Table 3. List of representative antibodies used in these studies.** A selection of the antibodies and the dilution factors used for aggregate immunostaining.

## Discussion

The technique described in this manuscript efficiently and reproducibly generates aggregates of mouse embryonic stem cells (mESCs) that display organized symmetry-breaking and elongation^19^, combining both the hanging drop culture described by Marikawa *et al.*^14^ and EB formation. These aggregates go on to develop axial elongations, complementing existing methods for the generation of anterior organs from ES cells such as optic cups^11^ and the cerebral cortex^13^, and contain cells with identities of the three germ layers which display processes similar to those during gastrulation such as rapid cell movement^19,21^.

It is interesting to speculate on the nature of the symmetry-breaking event, since it occurs in the absence of external patterning tissues and zygotic asymmetry cues^19^. The spontaneous formation of a rudimentary axis could arise from pre-existing heterogeneities in the starting culture of cells, which form the basis for the development of asymmetry. Indeed, populations of cells cultured in ESLIF conditions display a mixture of self-renewing and differentiating cells, the relative proportions of which may vary from one aggregate to another. Furthermore, preliminary work in our laboratory suggests that aggregates from a wholly pluripotent population of cells show delayed development and defects in patterning, suggesting a role for heterogeneity in organized pattern formation (D.A. Turner & A. Martinez Arias, in preparation). It is also worth considering that a reaction-diffusion mechanism may generate the pattern, with diffusion of an inhibitor away from the surface of the aggregate. Warmflash *et al.*^41^ suggest that such a mechanism could be responsible for developing radial asymmetry in adherent culture, making it a possible candidate for patterning in three dimensions.

During the initial 48 hr aggregation period, cells reach a state similar to that within the postimplantation epiblast, in which they are competent to respond to signals from the secondary medium^19,21,24^. Typically, the protocol described here provides cells with a pulse of secondary medium which contains factors (such as Wnt/β-Catenin agonists) that provide the signals sufficient for elongation. Previous culture methods described by Lancaster *et al. *(using human ESCs^13^) and Eiraku *et al. *(with mESCs^11^) used a short period of aggregation in low-adhesion 96-well plates before aggregated cells were transferred to Matrigel droplets for on-going culture. Although the time between aggregation and Matrigel insertion was different between the studies, in both cases, large numbers of cells were used for aggregate formation (approximately 3,000-4,500 cells). By contrast, the protocol described here^19^ uses far fewer cells (approximately 400 cells per aggregate) which has been determined to be absolutely essential to the process of elongation, symmetry-breaking and the polarization that is observed^19^. In addition, these elongated structures are able to be generated in a much shorter time-period without embedding in artificial matrices: compare the generation of defined brain regions by Lancaster *et al.* (>20-30 days)^13^ and the optic cups from Eiraku *et al.* (~11 days)^11^ with the 5 days required for the generation of polarized elongated structures.

One of the limitations with the culture method described here however, is that they can only be cultured for approximately 5-6 days post plating until their size renders them competent under gravity to sink to the bottom of the wells and force an adhesion even on non-coated plastic-ware. Further experiments using artificial matrices such as those mentioned previously, may allow us to increase the period of observation and experimentation, and work is on-going to determine the effects of constraining the aggregates in such a manner. A further limitation of this technique is that in order to change the medium without removing the aggregates, a small volume of medium must be left in the bottom of the well. The consequences for chemical genetics approaches are the need to consider this dilution and any residual effects from previous media: on the day of the 3 µM Chi pulse, 2.37 µM solution is actually delivered, and subsequent medium changes result in a carry-over of 0.499 µM (72-96 hr) and 0.105 µM (96-120 hr) between the time-points. Current work has not corrected for dilution and it is assumed that daily medium changes will minimize residual effects.

There are a number of critical steps in the protocol to ensure both intra- and inter-experimental reproducibility. Firstly, the starting culture of mESCs must be well maintained and not over confluent, and medium should always be good quality *i.e*., fresh and well mixed (**Figure 5A,B**). Poor culture conditions adversely affect the aggregation (**Figure 5Bi**). Accurate counting of cells to obtain a ~400 cells/40 µl cell suspension is essential, as larger aggregates fail to self-organize and are more likely to form double aggregates within each well (**Figure 5Bii**) whereas smaller aggregates are unable to reach the correct density where they are able to respond to the stimulus (**Figure 5Biii**). A key optimisation step was the inclusion of a second PBS wash that removes contaminant serum from the cell suspension (Step 2.6); this improved the aggregation frequency and accelerated the development of the aggregates by approximately 24 hr. Next, ensuring medium changes are applied with sufficient force to dislodge any aggregates that have the potential to adhere to the surface of the well; failure to do so results in the aggregates ‘crashing’ (**Figure 5Biv**). In addition, and as mentioned previously (and below), it is essential to ensure that the aggregates are maintained in suspension culture and are prevented from adhering to any surface. Once the aggregates have adhered, the pattern of gene expression and their elongation potential are disrupted.

As this technique is relatively straightforward, and well within the remit of individuals with good tissue-culture techniques, most of the problems associated with aggregation can be resolved relatively rapidly if these critical steps are followed; a full table of trouble-shooting is available (**Table 1**). Once mastered, this technique can be used to study axial development, symmetry breaking and cell-decision events. More specifically, these aggregates have the potential to generate pools of cells that have hitherto been unavailable in culture, such as the spinal cord and motor neurones.

**Ethical Note: **It should be noted with due caution that the translation of this protocol to human ES or iPS cell culture could bring the experimenter close to the legal basis of the fourteen day limit on human embryo research, namely the generation of a primitive streak, as detailed in the Human Fertilisation and Embryology Act 2008 (UK)^42^. Since the Act covers all live human embryos regardless of their manner of creation, culture of human gastruloids beyond the primitive-streak like stage would be beyond the scope of licensable research.

## Disclosures

The authors declare that they have no competing financial interests.
